# Exacerbations and *Pseudomonas aeruginosa* colonization are associated with altered lung structure and function in primary ciliary dyskinesia

**DOI:** 10.1186/s12887-020-02062-4

**Published:** 2020-04-13

**Authors:** G. Piatti, M. M. De Santi, A. Farolfi, G. V. Zuccotti, E. D’Auria, M. F. Patria, S. Torretta, D. Consonni, U. Ambrosetti

**Affiliations:** 1 Department of Pathophysiology and Transplantation, University of Milan and Unit of Bronchopneumology, Fondazione IRCCS Ca’ Granda Ospedale Maggiore Policlinico, Via Francesco Sforza 35 -, 20122 Milan, Italy; 2 Department of Human Pathology and Oncology, University of Siena and Unit of Pathological Anatomy, Policlinico Le Scotte, Strada delle Scotte 6, Siena, Italy; 3 Pediatric Pulmonology, Pediatric Department, Vittore Buzzi Children’s Hospital, University of Milan, via Castelvetro 32, 20154 Milan, Italy; 4 Department of Pathophysiology and Transplantation, University of Milan and Paediatric Highly Intensive Care Unit, Fondazione IRCCS Ca’ Granda Ospedale Maggiore Policlinico, Via Francesco Sforza, 35 Milan, Italy; 5 Department of Clinical Sciences and Community Health, University of Milan and Division of Otolaryngology, Fondazione IRCCS Ca’ Granda, Ospedale Maggiore Policlinico, Via Francesco Sforza 35, Milan, Italy; 6 Epidemiology Unit, Fondazione IRCCS Ca’ Granda Ospedale Maggiore Policlinico, Via Francesco Sforza, 35 Milan, Italy; 7 Department of Clinical Sciences and Community Health, University of Milan and Audiology Unit, Fondazione IRCCS Ca’ Granda Ospedale Maggiore Policlinico, Via Francesco Sforza, 35 Milan, Italy

**Keywords:** Primary ciliary dyskinesia, Respiratory exacerbations, *Pseudomonas aeruginosa* colonization, Chest CT scores

## Abstract

**Background:**

Recurrent bacterial infections of the respiratory tract are one of the major clinical features of the primary ciliary dyskinesia (PCD), a rare genetic disease due to malfunctioning of motile cilia. Chronic infections and persistent inflammation of the respiratory system result in progressive lung disease.

Aim of the study was to highlight the main factors associated with clinical, functional and anatomical deterioration in PCD patients.

**Methods:**

We retrospectively analyzed data from 58 patients with PCD, 37 adults and 21 children. The demographic and clinical data, forced expiratory volume at 1 s (FEV_1_) and forced vital capacity (FVC), sputum microbiology and imaging results (chest CT scores-modified Bhalla) were recorded. Patients were stratified according to the number of exacerbations (< 2/year vs ≥ 2/year) and chronic *Pseudomonas aeruginosa* (PA) colonization. The possible correlations between lung function and chest CT scores were assessed; we also evaluated the correlation between these parameters and the severity scores for bronchiectasis (BSI, FACED and e-FACED).

**Results:**

Chest CT scores showed a significant correlation with FEV_1_ (*p* = 0.0002), age (*p* <  0.0001), BMI (p = 0.0002) and number of lung lobes involved (p <  0.0001). PA colonization had an overall prevalence of 32.6%: no significant difference in FEV_1_ between PA colonized and non-colonized patients was found (*p* = 0.70), while chest CT score was significantly worse in chronic PA colonized patients (*p* = 0.009). Patients with a high number of exacerbation (≥ 2/year) were older (*p* = 0.01), had lower FEV_1_ (*p* = 0.03), greater number of lobes involved (*p* < 0.001) and worse CT score than patients with low number of exacerbations (*p* = 0.001); they also had higher prevalence of PA chronic bronchial infection (33.3% versus 13.6%, *p* = 0.10). Multivariable linear regression analyses adjusted for gender, age and BMI showed positive associations between PA colonization and number of exacerbations with severity of disease (number of lobes involved, CT score, BSI, FACED, and e-FACED).

**Conclusions:**

In our PCD population the number of exacerbations (≥ 2/year) and PA colonization were the two most relevant factors associated with severity of disease.

## Background

Primary ciliary dyskinesia (PCD, MIM #244400; http://www.ncbi.nlm.nih.gov/Omim/) is a rare, genetic and clinically heterogeneous disease, characterized by disorder of motile cilia in structure or function, resulting in chronic upper and lower respiratory tract disease, left-right laterality defect in approximately 50% of cases and infertility.

The disease has been described in 1933 by Kartagener as a triad of chronic sinusitis, bronchiectasis and situs viscerum inversus [1]. Afzelius in 1976 reported that these patients have immotile cilia and defective ciliary ultrastructure [2].

A prevalence of approximately 1 in 15,000 newborns has been estimated [3]. Age at presentation ranges from birth to adulthood. In the newborns, the clinical presentation of PCD is characterized by occurrence of respiratory distress in > 80% of cases [4], daily nasal congestion and wet cough starting soon after birth; recurrent or chronic middle ear and sinus disease develop early and most PCD patients demonstrate severe pansinusitis on computed tomography (CT) scan [5].

Recurrent pneumonia or bronchitis are very common in PCD, and in children with recurrent respiratory infections the prevalence of PCD can be as high as 5% [6].

Bronchiectasis may be present already in children with PCD, affect primarily the middle lobe, lingula and lower lobes and are age-dependent [4, 5]. As the airway disease does progress with age, consolidative areas, atelectasis and bronchiectasis become constant findings in adults [7].

There is no a pathognomonic test for the diagnosis of PCD. Therefore, a panel of tests, including nasal nitric oxide (nNO), transmission electron microscopy (TEM), evaluation of ciliary motility and genetic analysis, is often required for a final diagnosis of PCD [8]. The clinical features for patients who must be referred to diagnostic tests for PCD are reported by European Respiratory Society guidelines in 2017 [9].

Respiratory infections are major determinants of morbidity and mortality in patients with PCD, as they are correlated to a poor quality of life [10] and to functional decline [11]. *Haemophilus influentiae* and *Streptococcus pneumoniae* appear to be the most common pathogens isolated from patients with PCD during early childhood [4, 12]. In adult patients sputum cultures during exacerbations yield pathogens such as *Haemophilus influentiae*, *Staphylococcus aureus*, *Streptococcus pneumoniae* and *Pseudomonas aeruginosa* (PA) [13].

Exacerbations in children and adults with PCD, defined as an acute increase in respiratory symptoms [14], characterize the natural history of the disease.

During exacerbation, PCD patients clinically present a significant reduction in FEV_1_ and approximately 25% of children with PCD fails to recover to baseline lung function after treatment of a pulmonary exacerbation with intravenous antibiotics [15].

Although the severity of exacerbations has been correlated to the decline of lung function in adult patients with non-CF bronchiectasis [16], the effect of the number of exacerbations on the disease severity in patients with PCD has not yet investigated**.**

PA is an important pathogen in PCD, with an overall reported prevalence ranging between 27 and 35% [17]. In PCD the presence of PA colonization appears to increase with age [12, 17, 18].

Although it is well known that chronic infection with PA is associated with a decrease in lung function and an increased risk of death in patients with cystic fibrosis (CF) [19, 20], it is unclear to what extent PA colonization also contributes to the decline of lung function in PCD patients.

In this study we primarily aimed to evaluate if the number of exacerbations and PA colonization are associated with lung function decline and structural damage in a group of PCD patients.

## Methods

We retrospectively analysed data of 58 (21 children and 37 adults) clinical cases of PCD who have been diagnosed and followed up during the last 10 years at Center for Rare Diseases, Unit of Respiratory Diseases of the Policlinico Hospital, Milan. The Ethical Committee of the Hospital approved protocol and a written informed consent was obtained from the adult patients or parents of children.

Patients with a history suggestive for PCD were considered “PCD case” in presence of hallmark ciliary electron microscopy defect [21, 22], and/or in case of identification of non-ambiguous biallelic mutations in known PCD-associated genes (patients “PCD positive”) [7, 23]. Patients with a clinical suspicious of PCD who do not fulfill at least one of the above mentioned criteria to make a certain diagnosis (e.g patients with normal cilia ultrastructure at TEM and/or patients with no evidence of unambiguous biallelic mutations in known PCD-associated genes) were considered as “highly likely PCD” by the result of the combination of other diagnostic test, e.g. repeated low levels of nasal nitric oxide, and abnormal ciliary motility, according to ERS Guidelines [23].

Nasal nitric oxide (nNO) assessments were achieved in patients over 5 years from a chemiluminescence analyzer (CDL Ecomedics, Dürnten, Switzerland) using a single-breath online method at a constant flow rate of 50 mL/s. Values of nNO <  77 nL/min were considered as cut-off [24, 25].

Ciliary motility was studied on fresh respiratory epithelium (within 15 min) at 25 °C, under an Olympus BH-2 optical microscope equipped with Nomarsky interferential contrast and a heated stage; samples were obtained by cytology brushing on the nasal inferior turbinate. Ciliary beat frequency (CBF) (normal range: 11–20 Hz) [26] and ciliary beat pattern (CBP) were determined as previously published [27].

We also obtained samples of nasal mucosa by brushing for processing and observation at transmission electron microscopy (TEM). Samples of respiratory epithelium obtained by nasal brushing were fixed in 2.5% cacodylate-buffered glutaraldehyde, post-fixed in buffered 1% osmium tetroxide, dehydrated, embedded in resin; ultrathin sections were double-stained with uranyl-acetate and lead citrate and examined at 100 kV with a Philips 208S transmission electron microscope. At least 50 cross sections of cilia from different cells were observed in each specimen. Electron micrographs were taken at a magnification of X 110,000 to study the internal axonemal structure. The ultrastructural phenotype was defined as the main ultrastructural defect and the percentage of abnormal cilia among the total number of cilia was analysed, considering nonspecific defect when present up to 10% of cilia [21]. We considered hallmark diagnostic defects for PCD as reported by International Consensus guideline (BEAT PCD TEM Criteria) [28]; inner dynein arm (IDA) defects were not considered diagnostic except when apparent on repeated biopsies [22].

Genetic analysis was performed on genomic DNA extracted from peripheral blood according to standard protocols [29] and PCR amplified to study mutations in dynein genes using a DNA isolation kit (Roche, Milano, Italy); Next Generation Sequencing (NGS) was applied to study mutations on PCD-known genes (ACVR2B, CFC1, CRELD1, FOXH1, GJA1, LEFTY2, NKX2–5, NODAL, ZIC3, DNAH5, DNAI1, DNAI2, CCDC39, CCDC40, DNAH11, KTU/DNAAF2, LRRC50/DNAAF1, TXNDC3/NME8, DNAL1, INVS/HPHP2, DNAAF3, RSPH4A, RSPH9, OFD1, RGPR, CCDC103, HEATR2, HYDIN, LRRC6, DYX1C1) [23].

### General clinical data

Clinical anamnestic data were collected with special focus on the presence of upper and lower recurrent respiratory infections and on the number and severity of exacerbations; they were recorded during the routine visits of follow-up, usually occurring every three-four months.

In children older than 5 years of age, spirometry was performed according to the American Thoracic Society/European Thoracic Society (ATS/ERS guidelines) [30]. Results were expressed as a percentage of the predicted value for height and age; volumes and flows were considered as normal when > 80% of the expected value. Usually, each patient performed at least one spirometry per year; for the purpose of this assessment, the most recent examination was considered.

### Lower airways involvement

Diagnosis of bronchiectasis was based on criteria reported by Naidich et al. for chest CT scan [31]. For patients who had more than one chest CT scan, the more recent was taken into account to establish the severity of lung disease; anyway, no more than 6 months elapsed between chest CT scan and spirometry performing. The extent of bronchiectasis, severity of bronchial dilatation, bronchial wall thickness, presence of mucus plugging in large and small airways and decrease in parenchymal attenuation were scored for each lobe according to the modified Bhalla high-resolution computed tomography scoring system (mBhalla) [32]. Lingula was considered as separate lobe. If lobectomy had been performed a severity score of 3 was assigned to the missing lobe by arbitrary definition and distribution was presumed diffuse. The mean score for all lobes for each abnormality was calculated and lobar predominance was assessed. The CT scores ranged between 0 and 48.

In addition to mBhalla scoring system, BSI, FACED and e-FACED scores were also calculated, as multidimensional scoring systems created and validated to classify the severity of bronchiectasis [33–35]. BSI identifies patients at risk of future mortality, hospital admissions and exacerbations; FACED classifies the severity of bronchiectasis according to 5-years prognosis; e-FACED detects patients with more frequent exacerbations. Classification of severity was stratified into mild, moderate and severe according to the original Authors designations.

### Microbiological data

All available sputum cultures were analyzed: each patient had at least three sputum bacteriology per year; included patients had microbiological data for at least 1 year. Chronic bronchial infection was defined as the isolation of the same pathogen in sputum culture on two or more occasions, at least 3 months apart in a 1-year period [36]. Similarly, regarding PA colonization, we classified patients as non-colonized if they had never been cultured or cultured only once with this pathogen, and as colonized patients if they showed at least two positive sputum cultures for PA in 1 year (3 months apart) [18, 36].

### Number of exacerbations

We considered the definition of exacerbation in PCD as indicated by expert consensus (14). As the median of exacerbations was 2 per year prior to the analysis in our patients, we classified patients in two groups: Low-EXAC: < 2/year and High-EXAC ≥2/year.

### Statistical analysis

Mann-Whitney test and chi-square test were used to compare quantitative and categorical variables, respectively. We calculated Spearman’s rho correlation coefficient to evaluate the association between age, BMI, and indexes of anatomical and functional lung damage. We examined the relationship (slopes and 95% confidence intervals (CI)) between the dependent variables FEV_1_, FVC, number of lung lobes, chest CT score, BSI, FACED and e-FACED and the independent variables gender (males versus females), age (in 10 years), BMI (Kg/m^2^), PA colonization (yes/no) and exacerbation frequency (≥2 versus < 2) using univariate and multivariable linear regression models. Statistical analyses were performed with Stata 15 (StataCorp. 2017).

## Results

The study included 58 patients admitted between 2007 and 2017: 33 males and 25 females; 37 adults, mean age 39.4 years (range: 19–70), 21 children, mean age 11.1 years (range: 2–17).

A “positive PCD diagnosis” was met by 51 out of the 58 patients; a “highly likely PCD diagnosis” was met by 7 patients who had clinical history suggestive for PCD, low nasal nitric oxide in more occasions, and repeated abnormal ciliary motility; in these subjects genetic tests resulted negative, but all showed situs inversus and five had bronchiectasis at chest CT.

Demographic and clinical characteristics of all patients are summarized in Table 1.

**Table 1:** *Characteristics of patients reported as number and percentage or mean ± SD*

	Children	Adults
**Number of patients***(%)*	**21***(36.2)*	**37***(63.8)*
**PCD positive**	**18***(85.7)*	**33***(89.2)*
**PCD highly likely**	**3***(14.3)*	**4***(10.8)*
**Males***(%)*	**7***(33.3)*	**19***(51.3)*
**Age in years***(mean ± SD)*	11.1 ± 4.6	39.4 ± 14.4
**Age at diagnosis***(mean ± SD)*	4.3 ± 4.2	26.5 ± 16.2
**Situs inversus***(%)*	**14***(66.6)*	**22***(59.4)*
**Situs solitus***(%)*	**6***(28.6)*	**15***(40.5)*
**Situs ambiguous***(%)*	**1***(4.8)*	**0***(0.0)*
**Lobectomy***(%)*	**1***(4.8)*	**5***(13.5)*
**Percentage of predicted value**		
**FEV**_**1**_*(mean ± SD)*	93.3 ± 18.7	72.5 ± 23.4
**FVC***(mean ± SD)*	103.5 ± 15.6	85.2 ± 21.7
**Exacerbations***(mean ± SD)*	1.6 ± 1.0	2.3 ± 1.1
**Exacerbations ≥ 2/year***(%)*	**10***(47.6)*	**26***(70.3)*
**Colonization by**:		
***Pseudomonas aeruginosa***	**3***(14.3)*	**12***(32.4)*
**Other pathogens**	**9***(42.8)*	**9***(24.3)*
**Chest CT score** (mBhalla)	2.6 ± 6.2	16.2 ± 9.5
**Bronchiectasis***(%)*	**6***(28.6)*	**32***(86.5)*
**Lobes***(mean ± SD)*	0.6 ± 1	2.5 ± 1.3
**Severity of bronchiectasis**		
**BSI**	2.5 ± 3.1	5.1 ± 4.3
**FACED**	0.4 ± 0.8	1.2 ± 1.2
**eFACED**	0.8 ± 1.4	2.1 ± 2.0

Table 2 shows the results of diagnostic tests for PCD.

**Table 2:** Results of the diagnostic testing

	All *(%)*	Children *(%)*	Adults *(%)*
**nNO** (nL/min)	**45***(100)*	**13***(100)*	**32***(100)*
**Low***(< 77)*	**34***(75.6)*	**11***(84.6)*	**23***(31.9)*
**Normal***(≥ 77)*	**11***(24.4)*	**2***(15.4)*	**9***(28.1)*
**Ciliary motility**	**50***(100.0)*	**17***(100.0)*	**33***(100.0)*
**Complete immotility**	**11***(22.0)*	**1***(5.9)*	**10***(30.3)*
**Reduced CBF** (< 5 Hz)	**22***(44.0)*	**9***(52.9)*	**13***(39.4)*
**Reduced CBF** (5–10 Hz)	**4***(8.0)*	**1***(5.9)*	**3***(9.1)*
**Within normal range** (> 10 Hz)	**2***(4.0)*	**1***(5.9)*	**1***(3.0)*
**Absence of cilia**	**11***(22.0)*	**5***(29.4)*	**6***(18.2)*
**Ciliary beat pattern**	**50***(100.0)*	**17***(100.0)*	**33***(100.0)*
**Complete immotility**	**11***(22.0)*	**1***(5.9)*	**10***(30.3)*
**Minimal residual movement**	**12***(24)*	**5***(29.4)*	**7***(21.2)*
**Extremely stiff**	**8***(16)*	**2***(11.8)*	**6***(18.2)*
**Reduced proximal bending**	**6***(12)*	**3***(17.6)*	**3***(9.1)*
**Circular beating cilia**	**2***(4)*	**1***(5.9)*	**1***(3)*
**Absence of cilia**	**11***(22.0)*	**5***(29.4)*	**6***(18.2)*
**TEM ultrastructure**	**54***(100.0)*	**19***(100.0)*	**35***(100*.0)
**Only ODA absence**	**12***(22.2)*	**2***(10.5)*	**10***(28.6)*
**ODA + IDA absence**	**10***(18.5)*	**1***(5.3)*	**9***(25.7)*
**ODA + IDA absence + CC defect**	**2***(3.7)*	**1***(5.3)*	**1***(2.9)*
**IDA absence + MTD**	**12***(22.2)*	**9***(47.4)*	**3***(8.6)*
**CC defect**	**6***(11.1)*	**2***(10.5)*	**4***(11.4)*
**Only IDA absence**	**3***(5.6)*	**0***(0)*	**3***(8.6)*
**Normal**	**9***(16.7)*	**4***(21)*	**5***(14.2*)
**Genetic analysis**	**42***(100.0)*	**15***(100.0)*	**27***(100.0)*
**No mutations **	**16***(38.1)*	**6***(40.0)*	**10***(37.0)*
**DNAH5** **Mutations**	**15***(35.7)*	**5***(33.3)*	**10***(37.0)*
**DNAH11** **Mutations**	**6***(14.3)*	**2***(13.3)*	**4***(14.9)*
**DNAI1** **Mutations**	**3***(7.1)*	**1***(6.7)*	**2***(7.4)*
**Other** **mutations**	**2***(4.8)*	**1***(6.7)*	**1***(3.7)*

**ODA** = *Outer Dynein Arm*; **IDA** = *Inner Dynein Arm*; **CC** = *Central Complex;***MTD***= Microtubular disorganization*

The diagnosis of PCD was made at a mean age of 26.5 years in adults and 4.3 years in pediatric patients.

Body mass index (BMI) was significantly correlated with the age of subjects (n. 58, rho = 0.71, *p* < 0.0001).

Chest CT scan were available for 56 patients: the main findings in our patients with PCD included bronchiectasis, atelectasis, mucus plugging, peribronchial thickening and air trapping.

CT scan showed bronchiectasis in 32/37 (86.5%) of adults and in 6 (28.6%) of children, most often cylindrical bronchiectasis. The mean number of lobes involved: 1.8 ± 1.5, with a significant correlation with age (n. 56, rho = 0.62, *p* < 0.0001). The lung bases were more frequently involved than the upper lobes; the middle lobe and the lingular segment showed the most severe lesions.

Chest CT score (mBhalla) correlated with age (n. 56, rho = 0.63, *p* < 0.0001), BMI (n. 56, rho = 0.48, *p* = 0.0002), FEV_1_ (n. 45, rho = − 0.53, p = 0.0002) and number of lobes involved (n. 56, rho = 0.96, *p* < 0.0001).

No correlation was found between mBhalla score and age of diagnosis or between FEV1 and age of diagnosis.

As showed in Table 3, we found moderate to strong positive correlations between indexes of structural pulmonary impairment and substantial negative correlations between functional and anatomical indexes: in particular, lung structural damage measured by mBhalla score and the number of lung lobes involved are related to decreased respiratory function, considering FEV1 and FVC deviation from predictive value.

**Table 3:** Correlation (Spearman’s rho coefficients) between indexes of anatomical and functional lung damage

	Number of lung lobes	mBhalla	BSI	FACED	e-FACED	FEV_**1**_
**mBhalla**	0.94					
**BSI**	0.64	0.62				
**FACED**	0.70	0.64	0.84			
**e-FACED**	0.74	0.69	0.92	0.92		
**FEV**_**1**_	- 0.50	- 0.55	- 0.48	- 0.51	- 0.52	
**FVC**	- 0.40	- 0.45	- 0.46	- 0.37	- 0.48	0.77

***n*** = 43; **All*p*-values** < 0.02

Concerning the respiratory function, FEV_1_ was significantly correlated with age (n. 46, rho = − 0.38, *p* = 0.01). Instead, no relationship was observed between respiratory function parameters and ultrastructural phenotype and/or genotype.

Table 4 shows the distribution of subjects stratified according to the multidimensional severity scores for bronchiectasis: BSI, FACED, e-FACED; according to these scores, most patients were classified as mild or moderate in severity, although, especially BSI, found some subjects as severe.

**Table 4:** Severity of PCD patients according to multidimensional indexes for bronchiectasis

	Children (n)	Adults (n)
**Patients**	**16**	**34**
**BSI***(%)*		
**Mild 0–4**	**12***(75.0)*	**21***(61.8)*
**Moderate 5–8**	**3***(18.8)*	**5***(14.7)*
**Severe ≥ 9**	**1***(6.2)*	**8***(23.5)*
**FACED***(%)*		
**Mild 0–2**	**15***(93.8)*	**31***(91.2)*
**Moderate 3–4**	**1***(6.2)*	**3***(8.8)*
**Severe 5–7**	**–**	**–**
**eFACED***(%)*		
**Mild 0–2**	**14***(87.6)*	**20***(58.9)*
**Moderate 3–4**	**1***(6.2)*	**11***(32.3)*
**Severe 5–7**	**1***(6.2)*	**3***(8.8)*

Chronic bacterial colonization was present in 12/21 (57.1%) children and in 21/37 (56.8%) adults subjects. Predominant pathogens were *Haemophilus influentiae* (36.9%), *Staphylococcus aureus* (15.2%), *Streptococcus pneumoniae* (13.0%), *Escherichia coli* (8.7%) and *Moraxella catharralis* (6.5%). No patients had *non-tuberculous mycobacteria*. In 10 cases, no pathogens were cultured in sputum. Microbiological data were missing or incomplete in 12 subjects. Fifteen subjects, 12 adults and 3 children, corresponding to 32.4 and 14.3% respectively, had a chronic colonization by *Pseudomonas aeruginosa*. The overall prevalence of PA colonization was 32.6%.

Patients with chronic PA colonization were older than noncolonized patients: 34.4 ± 16.1 years versus 27.4 ± 18.6 years (*p* = 0.10). PA colonized patients also show worse chest CT score (*p* = 0.009), while we did not observed differences in FEV_1_ values (*p* = 0.70) between colonized and non-colonized patients, probably due to the limited number of PA colonized patients in our study.

Patients with a high number of exacerbations (≥ 2/year) were older (*p* = 0.01) and had lower FEV_1_ (*p* = 0.03) compared to patients with few exacerbations (< 2/year). The number of lung lobes involved (*p* < 0.001) and worse mBhalla scores (*p* = 0.001) were also positively associated with the number of exacerbations. Finally, a high number of exacerbations (≥ 2/year) patients showed a trend of higher prevalence of PA chronic bronchial infection than low exacerbation (< 2/year) patients (33.3% versus 13.6%, p = 0.10) (Table 5).

**Table 5:** **.** Distribution of selected parameters (mean ± SD) according to the two groups of patients stratified by the number of exacerbations

	High number of exacerbations (≥ 2/year)	Low number of exacerbations (< 2/year)	*p*
**Age*(years)***	33.9 ± 18.4	21.4 ± 14.9	0.01
**BMI*(Kg/m***^***2***^***)***	21.6 ± 4.6	18.6 ± 4.2	0.02
**FEV1*(% of predicted value)***	73.7 ± 22.7	89.1 ± 24.2	0.03
**Number of lung lobes**	2.3 ± 1.4	0.9 ± 1.2	< 0.001
**Chest CT score*(mBhalla)*(0–48)**	14.8 ± 10.8	5.2 ± 7.1	0.001
**PA colonization*(%)***	33.3	13.6	0.10

In univariate analyses age, BMI, PA colonization and a high number of exacerbations (≥2/year) were associated with number of lung lobes involved, chest CT score and multidimensional scoring systems (BSI, FACED and e-FACED) (Table 6, upper part). In a multivariable analysis, after adjusting for age, sex and BMI, PA colonization was positively associated with BSI, FACED, and e-FACED, while a high number of exacerbations was positively associated with number of lung lobes involved, chest CT score, BSI, and e-FACED (Table 6, lower part).

**Table 6:** Relationship between dependent variables (FEV_1_, FVC, number of lung lobes, chest CT score, BSI, FACED and e-FACED) and selected characteristics. Coefficients and 95% confidence intervals (CI) calculated with univariate and multivariable linear regression models

**Crude slopes***(95% CI)*							
Independent variables	FEV_**1**_*(%) Predicted value*	FVC (%) *Predicted value*	Number of lung lobes	Chest CT score	BSI	FACED	e- FACED
**Gender***(males* vs *females)*	+ 8.9 (− 5.3, + 23.2)	+ 0.1 (− 13.2, + 13.3)	- 0.6 (− 1.4, + 0.2)	- 4.6 (− 10.3, + 1.0)	- 1.6 (− 4.0, + 0.7)	- 0.6 (− 1.2, + 0.05)	- 0.8 (− 1.9, + 0.3)
**Age***(10 years)*	- 5.4 (− 9.2, − 1.6)	- 5.1 (− 8.6, − 1.6)	+ 0.4 (+ 0.2, + 0.6)	+ 2.9 (+ 1.5, + 4.3)	+ 0.9 (+ 0.3, + 1.5)	+ 0.2 (+ 0.03, + 0.4)	+ 0.4 (+ 0.1, + 0.7)
**BMI***(Kg/m*^*2*^*)*	- 1.3 (− 2.9, + 0.3)	- 1.2 (− 2.7, + 0.4)	+ 0.2 (+ 0.1, + 0.2)	+ 1.0 (+ 0.4, + 1.5)	+ 0.3 (+ 0.1, + 0.6)	+ 0.1 (+ 0.03, + 0.2)	+ 0.2 (+ 0.1, + 0.3)
**PA colonization** Yes (# 15) vs No (# 43)	- 4.6 (− 19.9, + 10.7)	- 2.0 (− 16.0, + 12.0)	+ 1.0 (+ 0.1, + 1.9)	+ 7.9 (+ 1.8, + 14.0)	+ 4.2 (+ 1.9, + 6.5)	+ 1.3 (+ 0.7, + 1.9)	+ 1.7 (+ 0.6, + 2.8)
**Exacerbations** ≥ 2 (# 36) vs < 2 (# 22)	- 15.4 (− 30.3, − 0.5)	- 8,2 (− 22.6, + 6.1)	+ 1.4 (+ 0.7, + 2.6)	+ 9.6 (+ 4.2, + 15.0)	+ 4.4 (+ 2.3, + 6.6)	+ 0.9 (+ 0.2, + 1.5)	+ 1.8 (+ 0.7, + 2.8)
** Slopes adjusted for gender, age, BMI, PA colonization, and exacerbations***(95% CI)*							
**Independent variables**	**FEV**_**1**_*(%) Predicted value*	**FVC (%)***Predicted value*	**Number of lung lobes**	**Chest CT score**	**BSI**	**FACED**	**e- FACED**
**Gender***(males* vs *females)*	+ 10.4 (− 3.5, + 24.3)	+ 1.0 (− 12.4, + 14.4)	- 0.7 (− 1.3, + 0.1)	- 4.9 (− 9.7, − 0.1)	- 0.9 (− 2.8, + 1.0)	- 0.4 (− 0.9, + 0.1)	- 0.5 (− 1.5, + 0.4)
**Age***(10 years)*	- 4.9 (− 9.7, − 0.2)	- 4.9 (− 9.4, − 0.4)	+ 0.2 (0.00, + 0.4)	+ 1.9 (+ 0.2, + 3.6)	+ 0.4 (− 0.3, + 1.1)	+ 0.05 (− 0.1, + 0.2)	+ 0.1 (− 0.2, + 0.4)
**BMI***(Kg/m*^*2*^*)*	- 0.1 (− 2.0, + 1.7)	+ 0.03 (− 1.8, + 1.9)	+ 0.1 (+ 0.01, + 0.2)	+ 0.4 (− 0.3, + 1.0)	+ 0.1 (− 0.1, + 0.4)	+ 0.1 (− 0.01, + 0.1)	+ 0.1 (− 0.03, + 0.2)
**PA colonization** Yes (# 15) vs No (# 43)	+ 0.5 (− 14.1, + 15.1)	- 0.7 (− 14.6, + 13.3)	+ 0.4 (− 0.4, + 1.1)	+ 3.7 (− 1.7, + 9.1)	+ 2.8 (+ 0.6, + 4.9)	+ 1.0 (+ 0.4, + 1.6)	+ 1.1 (0.0, + 2.1)
**Exacerbations** ≥ 2 (# 36) vs < 2 (# 22)	- 10.4 (− 25.2, + 4.4)	- 4.7 (− 19.0, + 9.7)	+ 0.7 (+ 0.1, + 1.4)	+ 5.2 (+ 0.1, + 10.3)	+ 2.9 (+ 0.8, + 5.0)	+ 0.4 (− 0.2, + 0.4)	+ 1.1 (+ 0.1, + 2.1)

## Discussion

This single centre, retrospective, cross-sectional study performed in both adults and children PCD patients, demonstrated a correlation between functional respiratory impairment, expressed by FEV_1_ and FVC deviation from predicted values, and lung structural damage, expressed by the number of lung lobes involved and higher chest CT scores. Our results are in agreement with those reported by other Authors [37–39]. In addition, our study aimed to assess how much two relevant factors as the exacerbations number and chronic PA colonization are related to functional and structural decline in PCD patients.

The exacerbations in PCD, defined as an acute increase in respiratory symptoms [14], are predictors of severity of disease and poor quality of life [10] and negatively impact on lung function, as bacterial infections are associated with morbidity and mortality in these patients [13]. It was reported that about 25% of children with PCD fail to recover to baseline lung function within 3 months following a pulmonary exacerbation treated with intravenous antibiotics and may never regain pre-exacerbation spirometry [15]. In PCD patients, during exacerbation, Ratjien et al. [40] observed that FEV_1_ drops from its previous baseline around a median change of 22%; they also reported that during pulmonary exacerbations in PCD patients, sputum analysis demonstrated neutrophilic airway inflammation, high interleukin IL-8 sputum concentrations and increased neutrophil elastase activity that is associated with lung decline over time, while neutrophil elastase decreased after antibiotic therapy.

In non-FC bronchiectasis patients the number of exacerbations is one of the major markers of disease activity and it is strictly related to prognosis in the short and long term [16]: the number and severity of exacerbations and the presence of PA chronic colonization are two factors both related with decline in lung function. Chalmers et al. [41] reported that in non-FC bronchiectasis patients, severe exacerbations were associated with more extensive bronchiectasis in terms of number of lobes involved or the presence of cystic bronchiectasis: for this reason, the identification of subjects at highest risk of functional decline is extremely important to avoid lung deterioration.

For the first time, our evaluation in PCD patients shows that the number of exacerbations (≥ 2/year) correlates with the severity of disease, in terms of structural damage and lung function severity of impairment: our data suggest that PCD patients with more than 2 exacerbations per year require a more careful surveillance to reduce and prevent a worsening of their respiratory conditions.

In the present study, patients were examined mostly in the clinical stability phase, so the data on the number and severity of exacerbations were predominantly anamnestic; overall, exacerbations were reported as mild or moderate, and only in some case hospitalization was necessary. On the other hand, the study was not focused on the severity of exacerbations but rather on the importance of the number of exacerbations themselves.

About the results of microbiology of sputum, they resulted in agreement with the results of other studies that reported *Haemophilus influentiae* and *Pseudomonas aeruginosa* as the most common pathogens in PCD [12, 18].

We found a prevalence of chronic PA colonization of 14.2% in children and 32.4% in adults; our data are similar to those reported by Wijers et al. who found that prevalence of PA colonization increases with the age, especially after age 30 in PCD, as in CF patients [13].

Considering both pediatric and adult PCD patients, Cohen Cymberknoh [18] also reported an overall prevalence of PA colonization of about 27%; those colonized with PA were older and diagnosed at a later age. Similarly, we observed that chronic PA lung colonization in PCD was associated with older age.

Identification of PA has been considered a key determinant of bronchiectasis severity in three recently developed severity scoring systems: the Bronchiectasis Severity Index (BSI), the FACED and e-FACED score that are multidimensional scores combining clinical, microbiological and radiological variables to evaluate the prognosis and severity of bronchiectasis [33–35].

In our study, PA colonized patients show worse chest CT score, as well as patients with number of exhacerbations > 2/year; FEV_1_ did not differ between colonized and non-colonized patients, but this result may be attributed to the small number of patients with PA colonization in this study. However, although we have not carried out a longitudinal study, the rate of decline in FEV1 derived from age classes between the colonized and the noncolonized groups was similar to those reported previously by Cohen-Cymberknoh et al. [18] and by Davis et al. [42].

In the study of Finch [43] the chronic PA colonization has been demonstrated to have a negative prognostic impact on non-FC bronchiectasis: adult patients with bronchiectasis colonized by PA had a threefold higher risk of mortality and a sevenfold greater risk of hospital admission.

Our results address the importance of early identifying and treating PA colonization to avoid lung structural damage in PCD patients. We applied the most frequent definition used in non-cystic fibrosis bronchiectasis studies for bacterial colonization that is “two or more isolates of the same microorganism at least three months apart in one year” [36, 43]. It is possible that microbiological studies on PCD patients may apply more stringent criteria to stratify subjects.

## Conclusions

The limitations of our study are due to its retrospective nature which precludes the possibility of proving a clear causal relationship between the number of chest exacerbations and PA colonization with the lung structure and function decline, even if an association between these factors can be hypothesized. Our results would require confirmation in larger studies, possibly using a prospective cohort design. For the future, a larger international studies should be carried out to better identify predictors of bronchiectasis development and lung function worsening in patients affected by PCD. However, our study performed as single centre included a relatively high number of patients both children and adults patients affected by PCD, a rare disease.

The use of different scores of lung structural damage, especially FACED and e-FACED, are easy to apply in clinical practice, since they require knowledge of a few clinical variables related to patients and demonstrate to be useful in PCD subjects for the stratification of the disease severity, rather than performing the more complex evaluation of the extent of lesions at chest CT, mainly used for research purposes.

Our data also underline the possibility to stratify PCD patients according to the aforementioned risk factors (≥ 2 exacerbations/year and PA colonization) and to intensify treatment of the airway infections at the first sign of worsening respiratory symptoms to prevent lung function and structure deterioration.

## Data Availability

The datasets used and/or analysed during the current study are available from the corresponding author on reasonable request.

## Abbreviations

**BMI**: Body mass index
**BSI**: Bronchiectasis Severity Index
**CBF**: Ciliary beat frequency
**CBP**: Ciliary beat pattern
**CC**: Central Complex
**CF**: Cystic fibrosis
**FEV1**: Forced expiratory volume at 1 s
**FVC**: Forced vital capacity
**mBhalla**: Modified Bhalla
**IDA**: Inner dynein arm
**MTD**: Microtubular Disorganization
**nNO**: Nasal nitric oxide
**ODA**: Outer dynein arm
**PA**: *Pseudomonas aeruginosa*
**PCD**: Primary ciliary dyskinesia.

## References

[1] Kartagener M (1933). Zur pathogenese der bronkiectasien. Bronkiectasien bei situs viscerum inversus. Beitr Klin Tuberk.

[2] Afzelius BA (1976). A human syndrome caused by immotile cilia. Science.

[3] Afzelius BA (1988). Immotile cilia syndrome: past, present and prospects for the future. Thorax.

[4] Noone PG, Leigh MW, Sannuti A, Minnix SL (2004). Primary ciliary dyskinesia: diagnostic and phenotypic features. Am J Resp Crit Care Med.

[5] Knowles MR, Daniels LA, Davis SD, Zariwala MA (2013). Primary ciliary dyskinesia: recent advances in diagnostics, genetics and characterization of clinical disease. Am J Resp Crit Care Med.

[6] Chapelin C, Coste A, Reinert P, Boucherat M, Millepied MC et a. Incidence of PCD in children with recurrent respiratory diseases. Ann Otol Rhinol Laryngol 1997;106:854–858. 10.1177/000348949710601008 9342982

[7] Werner C, Onnebrink JG, Omran H (2015). Diagnosis and management of primary ciliary dyskinesia. Cilia.

[8] Shapiro AJ, Zariwala MA, Ferkol T (2016). Diagnosis, monitoring and treatment of primary ciliary dyskinesia: PCD Foundation consensus recommendations based on state of art review. Pediatr Pulmonol.

[9] Lucas JS, Barbato A, Collins SA (2017). European Respiratory Society guidelines for the diagnosis of primary ciliary dyskinesia. ERJ.

[10] Nathan AM, De Bruyne JA, Peng Eg K, Thavagnanam S (2017). Review: quality of life in children with non-cystic fibrosis bronchiectasis. Front Pediatr.

[11] Santamaria F, Montella S, Tiddens AWM, Guidi G (2008). Structural and functional lung disease in primary Ciliary dyskinesia. Chest.

[12] Alanin MC, Wijers CDM, Nielsen KG, von Buchwald C, Skov M (2015). A longitudinal study of lung bacterial pathogens in patients with primary ciliary dyskinesia. Clin Microbiol Infect.

[13] Wijers CDM, Chmiel JF, Gaston BM (2017). Bacterial infections in patients with primary ciliary dyskinesia: comparison with cystic fibrosis. Chronic Resp Dis.

[14] Lucas JS, Gahleitner F, Amorim A, Boon M (2019). Pulmonary exacerbations in patients with primary ciliary dyskinesia: an expert consensus definition for use in clinical trials. ERJ Open Res.

[15] Sunter M, Bush A, Hogg C, McCann L, Carr SB (2016). Recovery of baseline lung function after pulmonary exacerbation in children with PCD. Pediatr Pulmonol.

[16] Martinez-Garcia MA, Soler-Cataluna JJ, Perpina-Tordera M, Roman-Sanchez P, Soriano J (2007). Factors associated with lung function decline in adults patients with stable non-cystic bronchiectasis. Chest.

[17] Chang H, Adjemian J, Dell SDM (2015). Prevalence of airway microbial flora in primary ciliary dyskinesia. Am J Respir Crit Care Med.

[18] Cohen Cymberknoh M, Weigert N, Gileles-Hillel A, Breuer O (2017). Clinical impact of Pseudomonas aeruginosa colonization in patients with primary Ciliary dyskinesia. Resp Med.

[19] LiPuma JJ (2010). The changing microbial epidemiology in cystic fibrosis. Clin Microbiol Rev.

[20] Emerson J, Rosenfeld M, McNamara S (2002). Pseudomonas aeruginosa and other predictors of mortality and morbidity in young children with cystic fibrosis. Pediatr Pulmonol.

[21] Papon JF, Coste A, Roudot-Thoraval FR (2010). A 20-year experience of electron microscopy in the diagnosis of primary ciliary dyskinesia. Eur Resp J.

[22] O’Callaghan C, Rutman A, Williams GM, Hirst RA (2011). Inner dynein arm defects causing primary ciliary dyskinesia: repeat testing required. Eur Resp J.

[23] Kuehni CE, Lucas JS (2017). Diagnosis of primary ciliary dyskinesia: summary of ERS task force report. Breathe.

[24] ATS/ERS recommendations for standardized procedures for the online and offline measurement of exhaled lower respiratory nitric oxide and nasal nitric oxide. Am J Resp Crit Care Med. 2005;171(8):912–930. 10.1164/rccm.200406-710ST 15817806

[25] Leigh MW, Hazucha MJ, Chawla KK (2013). Standardizing nasal nitric oxide measurement as a test for primary ciliary dyskinesia. Ann Am Thorac Soc.

[26] Jackson CL, Behan L, Collins SA (2016). Accuracy of diagnostic testing in primary ciliary dyskinesia. Eur Resp J.

[27] Piatti G, Ambrosetti U, Santus P, Allegra L (2005). Effects of salmeterol on cilia and mucus in COPD and pneumonia patients. Pharmacol Res.

[28] Shoemark A, Boon M, Brocchhausen C, Bukowy-Bieryllo Z et al. International consensus guideline for reporting transmission electron microscopy results in the diagnosis of primary Ciliary dyskinesia (BEAT PCD TEM criteria). ERJ 2020. 14 (in press). 10.1183/13993003.00725-2019 32060067

[29] Zuccarello D, Ferlin A, Cassadore C, Pepe A, Garolla A, Moretti A, Cordeschi G (2008). Mutations in dynein genes in patients affected by isolated non-syndromic asthenozoospermia. Hum Reprod.

[30] ATS/ERS Task force: standardization of lung function testing. Interpretative strategies for lung function test. ERJ. 2005;26:948–968. 10.1183/09031936.05.00035205 16264058

[31] Naidich DP, McCauley DI, Khouri NF, Stitik FP, Siegelman SS (1982). Computed tomography of bronchiectasis. J Comput Assit Tomogr.

[32] Bhalla M, Turcios N, Aponte V (1991). Cystic fibrosis: scoring system with thin sections CT. Radiology.

[33] Chalmers JD, Goeminne P, Aliberti S, McDonnell MJ (2014). The bronchiectasis severity index. An international derivation and validation study. Am J Respir Crit Care Med.

[34] Martinez-Garcia MA, DE Gracia J, Vendrell Relat M (2014). Multidimensional approach to non-cystic fibrosis bronchiectasis: the FACED score. Eur Resp J.

[35] Martinez-Garcia MA, Athanazio RA, Giron R, Maiz-Carro L (2017). Predicting high risk of exacerbations in bronchiectasis: the e-FACED score. Internat J COPD.

[36] Pasteur MCMC, Helliwell SM, Houghton SJ, Webb SC, Foweraker JE, Coulden RA (2000). An investigation into causative factors in patients with bronchiectasis. Am J Resp Crit Care Med.

[37] Shah A, Shoemark A, MacNeill SJ, Bhaludin B (2016). A longitudinal study characterising a large adult primary ciliary dyskinesia population. ERJ.

[38] Lémery-Magnin M, Cros P, Beydon N (2012). Longitudinal lung function and structural changes in children with primary Ciliary dyskinesia. Pediatr Pulmonol.

[39] Frija-Masson J, Bassinet L, Honoré I (2017). Clinical characteristics, functional respiratory decline and follow-up in adult patients with primary ciliary dyskinesia. Thorax.

[40] Ratjen F, Waters V, Klingel M, McDonald N (2016). Changes in airway inflammation during pulmonary exacerbations in patients with CF and PCD. ERJ.

[41] Chalmers JD (2017). Bronchiectasis: phenotyping a complex disease. COPD: J Chron Obstruct Pulmon Dis.

[42] Davis SD, Rosenfeld M, Lee HS, Ferkol TV (2019). Primary ciliary dyskinesia: longitudinal study of lung disease by ultrastructure defect and genotype. AJRCCM.

[43] Finch S, McDonnell MJ, Abo-Leyah H, Aliberti S (2015). A comprehensive analysis of the impact of PA colonization on prognosis in adult bronchiectasis. Ann Am Thorac Soc.

